# Impact of Obstructive Sleep Apnea (OSA) in COVID-19 Survivors, Symptoms Changes Between 4-Months and 1 Year After the COVID-19 Infection

**DOI:** 10.3389/fmed.2022.884218

**Published:** 2022-06-14

**Authors:** Gonzalo Labarca, Mario Henríquez-Beltrán, Liliana Lamperti, Estefania Nova-Lamperti, Sergio Sanhueza, Camilo Cabrera, Romina Quiroga, Barbara Antilef, Valeska Ormazábal, Felipe Zúñiga, Daniela Castillo, Gloria Horta, Daniel Enos, Jaime Lastra, Jessica Gonzalez, Adriano Targa, Ferran Barbe

**Affiliations:** ^1^Molecular and Translational Immunology Laboratory, Clinical Biochemistry and Immunology Department, Faculty of Pharmacy, Universidad de Concepción, Concepción, Chile; ^2^Division of Sleep and Circadian Disorders, Brigham and Women's Hospital and Harvard Medical School, Boston, MA, United States; ^3^Escuela de Kinesiología, Facultad de Salud, Universidad Santo Tomás, Los Ángeles, Chile; ^4^Departamento de Farmacología, Facultad de Ciencias Biológicas, Universidad de Concepción, Concepción, Chile; ^5^Division of Gastroenterology and Hepatology, Beth Israel Deaconess Medical Center and Harvard Medical School, Boston, MA, United States; ^6^Internal Medicine, Healthcare Complex Dr. Victor Rios Ruiz (CAVRR), Los Angeles, Chile; ^7^Internal Medicine, Faculty of Medicine, Universidad de Concepcion, Los Angeles, Chile; ^8^Translational Research in Respiratory Medicine (TRRM) Group, Hospital Universitari Arnau de Vilanova-Santa Maria, Biomedical Research Institute of Lleida (IRBLleida), Lleida, Spain; ^9^Centro de Investigación Biomédica en Red de Enfermedades Respiratorias (CIBERES), Madrid, Spain

**Keywords:** COVID-19, obstructive sleep apnea, symptoms, cytokines, neurocognitive impairment

## Abstract

**Objective:**

To determine the association between Obstructive Sleep Apnea (OSA) with long-term symptoms and inflammatory cytokines, exploring the changes between 4-months and 1-year after COVID-19 infection.

**Methods:**

We conducted an observational, prospective cohort study, including patients ≥18 years old with confirmed diagnosis of COVID-19 between April to July 2020. All participants underwent two clinical follow-up visits, the first at 4-months (Visit 1) and the second at 1 year, after SARS-CoV-2 infection (Visit 2). Plasma glucose, total cholesterol, HDL, and triglycerides. Regarding pulmonary function, spirometry and lung diffusion capacity tests were assessed. For mental and neurocognitive evaluation, a short-form (SF-12), Beck depression and Hospital-Anxiety depression questionnaires were conducted at both time-points, whereas the Montreal Cognitive assessment was conducted during the second follow-up. Regarding to sleep evaluation, Epworth Sleepiness Scale, Insomnia Severity index and STOP-BANG questionnaire were conducted. Additionally, a home sleep apnea test and 7-day wrist actigraphy were performed in all participants. Inflammatory cytokines were measured using an inflammatory cytokine bead array kit. *p-*values < 0.05 were considered statistically significant and statistical analyses were performed using R software.

**Results:**

A total of 60 patients were included in the first follow-up, from which 57 completed the second follow-up. The mean age was 46.4 years-old (SD ± 13.1) and 53.3% were male. 30% of cases reported mild COVID-19 infection, 28.3% with moderate illness, and 41.6% with severe illness. Moreover, 56.6% of them were admitted to the ICU. Regarding to metabolic values, the OSA group showed higher values of insulin resistance (IR) (27%), systolic blood pressure (SBP) 135.2 (±19.1), dyslipidemia (67.5%), total cholesterol 202.1 (±60.5), triglycerides 176.1 (±119.0) and HOMA-IR 9.0 (±18.8) in comparison with the non-OSA group. 1 year after COVID-19 infection, DLCO test remains abnormal in OSA patients (25% OSA vs. 3.6% non-OSA, *p* = 0.02). Finally, those participants with OSA who develop ARDS reported an adjusted OR 20.4 (95%-CI, 1.04–504) risk of neurocognitive impairment.

**Discussion:**

Among patients with previous COVID-19, OSA impact the development of incident glycemic, neurocognitive impairment, and abnormal functional pulmonary changes that persist up to 1 year since acute phase.

## Background

The Coronavirus 2019 (COVID-19) disease is an emergent disease secondary to the SARS-CoV-2 infection, with a major impact in health outcomes (1)^1^. During the acute period, there are different severities described (asymptomatic, mild, moderate, and severe COVID-19), including some cases of severe pneumonia and the development of acute respiratory distress syndrome (ARDS) (2–4). After the acute phase of COVID-19, there is a prolonged period of persistent symptoms associated to the disease, described as “long COVID-19” or “persistent COVID-19”. In addition, there are several sequalae described through the entire organism including pulmonary function, mental health disturbance, cardiometabolic dysregulation, and worse neurocognitive outcomes during the first year after the SARS-CoV-2 infection (5–7).

To date, most of the data published on the risk factors for the pneumonia in COVID-19 and long-term sequelae (as example: male gender, age, obesity, hypertension, diabetes) are also recognized as clinical risk factor for increased risk of obstructive sleep apnea (OSA) (8–11). However, data about the association between OSA and COVID-19 is limited to few studies, including indirect measures such as questionnaires, or secondary analysis of databases (1, 8, 12, 13).

OSA is associated to intermittent hypoxemia, sympathetic activation, and sleep fragmentation, with studies reporting a multisystemic impairment among untreated OSA (14, 15). Therefore, the interaction between COVID-19 and OSA should be explored to better identify the clinical impact of OSA and long-term symptoms. One previous study associated the presence of OSA with an independent risk of persistent abnormal Chest Computed Tomography (CT) at 4-months after the infection (16).

Herein, our hypothesis is that OSA is associated with an increased burden of symptoms 1 year after the infection. The aim of this study was to determine the association between OSA with long-term symptoms and inflammatory cytokines, exploring the changes between 4-months and 1 year after the COVID-19 infection.

## Materials and Methods

### Study Design

We conducted an observational, prospective cohort study following current recommendations from STROBE statement (17). The study protocol was previously registered in the ISRCTN registry (ID: ISRCTN16865246) and was approved by the Institutional Review Board (IRB) from Servicio de Salud Bio Bio (IRB: CEC113), and Servicio de Salud Concepcion (IRB: CEC-SSC: 20-07-26) in Chile. Signed informed consent was acquired prior to inclusion in the study, and all methods were performed in accordance with the Helsinki Declaration and Good Clinical Practice.

We included patients ≥18 years old with confirmed diagnoses of COVID-19 between April to July 2020. All cases were confirmed to be positive by SARS-CoV-2 PCR and reported antibodies IgM/IgG to SARS-CoV-2 4-months after the acute infection. We excluded patients who were lost to follow-up, transferred to another hospital or city after discharge, and in palliative care, persistent oxygen requirement or mechanical ventilation, decompensate chronic comorbidities and those with mental disability that might prevent them from completing the evaluations.

### Clinical Data

All participants underwent two follow-up clinical visits at 4-months (Visit 1) and 1 year after SARS-CoV-2 infection (Visit 2). All participants were clinically evaluated, obtaining the previous history of COVID-19-derived symptoms and severity (mild, moderate, severe, or critical illness) during the acute infection. Additionally, we extracted data about sociodemographic such as age, sex, education (<8 years; 8–12 years, >12 years), living area (rural/urban), tobacco history (current, former, or never smoker) and alcohol intake (frequently, occasionally, never). In addition, information about comorbidities at baseline such as arterial hypertension (HT), insulin resistance (IR), type 2 diabetes mellitus (T2DM), hypothyroidism, arrhythmia, coronary heart disease or stroke, and current medication, were collected through both self-report, and medical records.

According to current recommendations, blood pressure was measured with a standard mercury sphygmomanometer on the left arm after at least 10 min of rest (18). Weight and height were measured after the subjects had fasted overnight and were wearing only underwear. Body mass index (BMI) was calculated as weight (Kg)/height (m^2^). Neck, waist, and hip circumferences were measured using a plastic tape meter at the cricoid level, umbilicus, and greater trochanters, respectively, and waist to hip ratio (WHR) was calculated.

### Laboratory Data

All participants provided a venous blood sample in both visits. The sample was obtained in the morning after an overnight fast. We evaluated the following laboratory parameters: (1) plasma glucose using a glucose-oxidase method, and total plasma cholesterol, HDL cholesterol, and triglycerides were assessed with standard enzymatic spectrophotometric technique. Plasma LDL was calculated by the Friedewald equation (19). Plasma insulin was measured using a radioimmunoassay. Homeostasis model assessment-estimated insulin resistance (HOMA-IR) was calculated according to the method by Mathew et al. (20) (Fasting plasma glucose (mmol/l) times fasting serum insulin (mU/l) divided by 22.5). Additionally, a glycated hemoglobin (HbAC1) was included at Visit 2. In this study, incident diabetes was achieved in both visits, following current criteria from the American Diabetes Association (ADA) (21).

### Pulmonary Function Test

We performed a baseline and post-bronchodilator (15 min after 400 μg of albuterol) spirometry (CPF-S / D; Medical Graphics Inc., EE. UU.) (22). Additionally, a Diffusing capacity of the lungs for carbon monoxide (DLCO) corrected by hemoglobin (Elite Platinum DL, Medical Graphics Inc, EE. UU.) (23); and 6-min walk test (6MWT) (24), were performed in both visits, and all procedures followed the current recommendation of the American Thoracic Society (ATS). The protocol of each evaluation was previously published (5). An abnormal DLCO at 1 year was defined as DLCO(c) <80%.

### Mental and Neurocognitive Evaluation

In both visits, a short form-12 questionnaire (including physical and mental domains) (25), Beck depression questionnaire (26), and the Hospital- Anxiety depression (HADS) (27), questionnaires were conducted. Additionally, a neurocognitive evaluation trough the Montreal Cognitive assessment (MOCA) was performed in the second visit, and a value <25 was suggestive of neurocognitive impairment (28).

### Inflammatory Cytokines

IL-1β, IL-6, IL-8, IL-2 and TNF-α were measured in serum samples with the Human Inflammatory Cytokine Cytometric Bead Array (CBA) Kit (BD) in both time points. The kit is based on the measurement of several cytokines at the same time from the same samples, for this, it uses a method of capture of the analyte, either soluble or a set of analytes, from the use of beads of size and known fluorescence, which are conjugated to a specific antibody. For the binding of the capture bead and the analyte to be quantifiable, the detection reagent provided in the kit was used, which is a mixture of antibodies conjugated with phycoerythrin (PE), which provides a fluorescent signal in proportion to the amount of bound analyte. Samples were acquired on a LSR-Fortessa X20 (BD) and quantified with FCAP Array Software v3.0 (BD Biosciences).

### Sleep Evaluation

The following sleep-related questionnaires were completed by all participants: Epworth Sleepiness Scale (ESS), Insomnia by the International Severity index (29), and STOP-BANG questionnaire (30). A home sleep apnea test (HSAT) was performed using an Apnea Link Air (ResMed, Australia) in addition to a 7 days wrist actigraphy (condor Ins, Brazil), following the current recommendations and requirements of the American Academy of Sleep Medicine (AASM) for level III studies (31). The HSAT analysis was performed manually (32). The HSAT following variables of were also included: respiratory disturbance index (apneas or hypopneas associated with 3% oxygen desaturation per hour), mean oxygen saturation (mean SpO2), minimum oxygen saturation (min SpO2), total time with oxyhemoglobin saturation below 90% (T90%), and oxygen desaturation index (ODI-≥3%). From actigraphy we extracted data about Total Sleep Time (TST), sleep efficiency, and wake after sleep onset (WASO). OSA was defined by a Respiratory disturbance index (RDI) ≥5 ev/h, and moderate to severe OSA was defined by RDI ≥15 ev/h (33).

### Analysis Plan

The means (standard deviation, SD) and medians (interquartile range) were estimated for quantitative variables with normal and non-normal distributions, respectively. The absolute and relative frequencies were used for qualitative variables. The Shapiro-Wilk and *t*-test analyzed distribution normality. The appropriate tests were assessed to determinate differences between the OSA and non-OSA groups, based on the clinical variables, and these tests include *t*-test, chi-squared (for parametric variables), Mann–Whitney U-test (for non-parametric variables). Additionally, we performed a Wilcoxon matched pair signed rank test, or Fisher's exact test, to compare the changes in the AHI, T90%, ODI-3%, min SpO2, BMI, and Mental questionnaire between Visit 1 and Visit 2.

### Risk of Incident Outcomes at 1 Year

The association between OSA with the risk of incident IR, T2DM, uncontrolled hypertension (defined as a measure >140/90 mmHg at evaluation), abnormal DLCO, and neurocognitive impairment reported at visit 2, were evaluated. For IR and T2DM analysis, subjects with prevalent diabetes or insulin resistance at baseline and visit 1 were excluded. A logistic regression analysis, as confounder analysis, and an unadjusted an adjusted model by age, gender, BMI, development of ARDS during the acute infection were performed. The values were reported as Odds ratios (OR) with a 95% confidence interval (95%-CI).

### Exploratory Analysis

Interaction between OSA severity, gender, development of ARDS during the acute infection and development of incident outcomes were tested by exploratory analysis. All tests were two-tailed, and *p-*values < 0.05 were considered statistically significant. Statistical analyses were performed using R software.

## Results

### Cohort Characteristics

**Figure 1:**
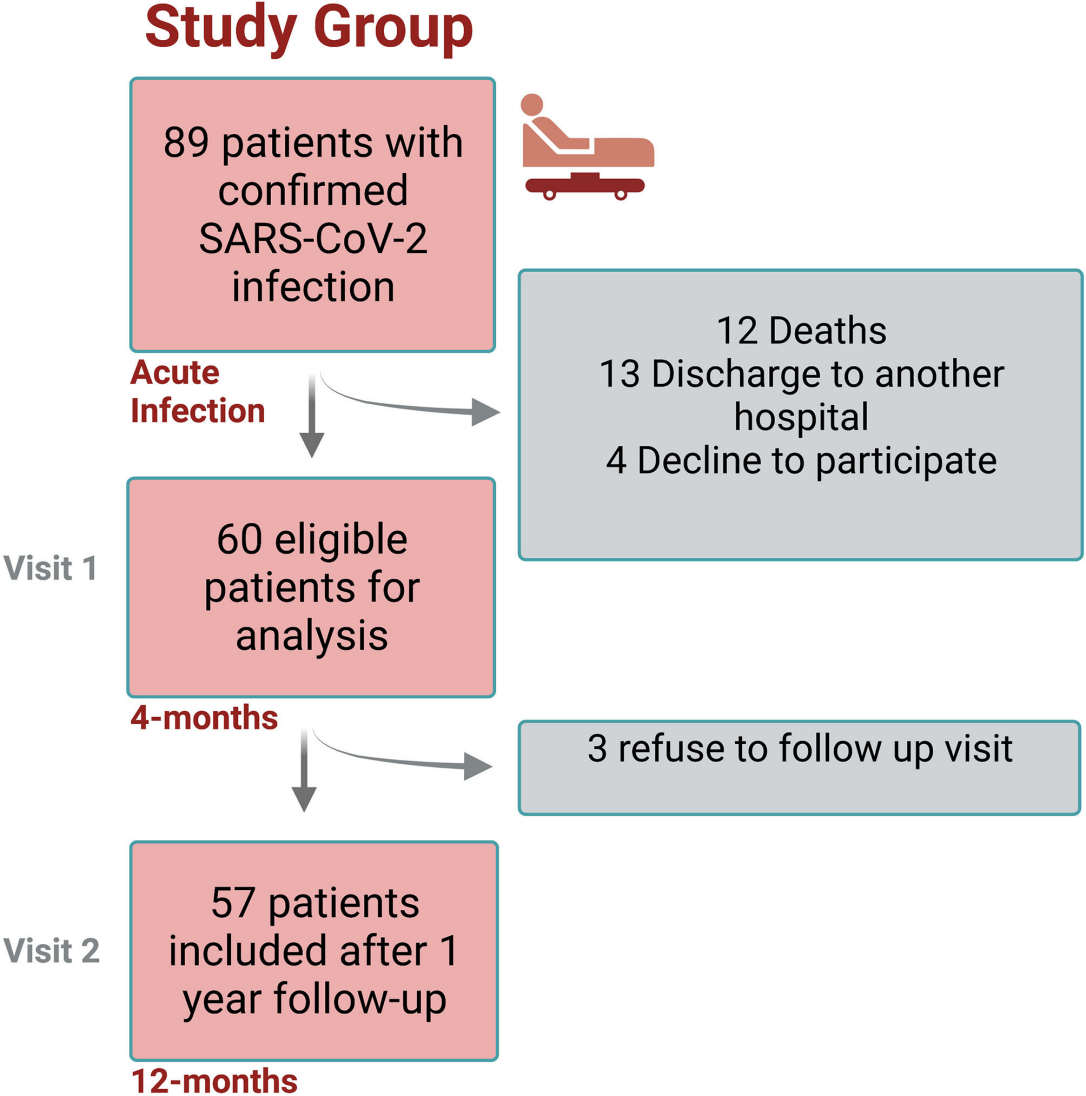
Study design flowchart.

The study design flowchart is shown in Figure 1. A total of 89 participants were invited to participate in study, and a total of 60 were included after informed consent (Visit 1), from which 57 completed Visit 2. The average age of the entire cohort was 46.4 years-old (SD ±13.1), mean follow-up since SARS-CoV2 infection to visit 1 was 16.2 weeks (±3.7), and 32/60 (53.3%) were male. At baseline (before COVID-19 infection), the prevalence of arterial hypertension was 19/60 (31.6%), 11/60 (18.3%) showed insulin resistance, and 7/60 (11.6%) had T2DM. According to CDC criteria, 18/60 (30%) cases reported to have a mild COVID-19 infection, 17/60 (28.3%) with moderate illness, and 25 (41.6%) with severe illness. Moreover, 34/60 (56.6%) of them were admitted to the ICU. A summary of baseline characteristics and ICU stay are shown in Table 1.

**Table 1:** Baseline characteristics of the complete cohort (*n* = 60).

**Variables**	
Sex, male, *n* (%)	32 (53.3%)
Age, years, mean (SD)	46.6 (±13.1)
**Scholar**	
<8 years, *n* (%)	21 (35%)
8–12 years, *n* (%)	15 (25%)
>12 years, *n* (%)	24 (40%)
Rural area, *n* (%)	6 (10%)
**Anthropometry**	
BMI, (K/m2), (SD)	31.0 (±4.9)
Neck circumference (cms), (SD)	41.8 (±5.1)
Waist circumference (cms), (SD)	103.6 (±12.6)
Hip circumference (cms), (SD)	108.5 (±9.3)
**Tobacco status**	
Current, *n* (%)	8 (13.3%)
Former, *n* (%)	14 (23.3%)
Never Smoker, *n* (%)	37(61.6%)
Alcohol status	
Occasionally, *n* (%)	12 (20%)
Frequently, *n* (%)	23 (38.3%)
Never, *n* (%)	25 (41.6%)
**Comorbidities**	
Arterial Hypertension, *n* (%)	19 (31.6%)
Insulin Resistance, *n* (%)	11 (18.3%)
Type 2 diabetes mellitus, *n* (%)	7 (11.6%)
Coronary heart disease, *n* (%)	2 (3.2%)
Hypothyroidism, *n* (%)	5 (8.3%)
Dyslipidemia, *n* (%)	12 (20%)
**Medications**	
ECA/ARA2, *n* (%)	12 (20%)
beta blockers, *n* (%)	3 (5%)
Ca blq, *n* (%)	6 (10%)
Aldosterone inhibitor, *n* (%)	1 (1.6%)
Diuretic drugs, *n* (%)	4(6.6%)
Metformin, *n* (%)	16 (26.6)
Insulin, *n* (%)	6 (10%)
Hipolipemiant drug, *n* (%)	12 (20%)

*18 (30%) cases reported to have a mild COVID-19 infection, 17 (28.3%) with moderate illness, and 25 (41.6%) with severe illness. Moreover, 34 (56.6%) of them were admitted to the ICU. BMI, Body mass index; SD, Standard deviation*.

### Prevalence of Undiagnosed OSA During the Follow-Up (Visit 1)

A summary of the respiratory sleep study test of the complete cohort is shown in Table 2. A total of 37/60 (61.6%) reported OSA. The average RDI was 16.0 ev/h (9.7). 22/37 (59.4%) reported mild OSA, 12/37 (32.4%) had moderate OSA, and 3/37 (8.1%) had severe OSA. Average ESS was similar between groups (9.56 ± 5.6 vs. 7.5 ± 4.8 points, *p* = 0.15). Then, when the clinical characteristic between OSA and non-OSA group were compared, we observed that 22/37 (59.4%) of the OSA group are male and 70.2% are current or former smokers. We also found significant differences regarding age, disease severity, STOP-BANG, rural area, neck and waist circumference and WHR ratio. Furthermore, despite similar BMI between groups, subjects with OSA reported increased baseline IR than the non-OSA group (Table 3).

**Table 2:** Respiratory sleep study test of the complete cohort (*n* = 60).

**Variable**	**Non OSA (*n =* 23)**	**OSA (*n =* 37)**	***p-*value**
Record (min), (SD)	339 (±133)	361 (±105)	0.5
RDI (ev/h), (SD)	2.6 (±1.4)	16.0 (±9.7)	*0.01
T90 (%), (SD)	1.4 (±4.6)	12.2 (±20.8)	*0.01
ODI (ev/h), (SD)	2.5 (±2.0)	15.1 (±11.6)	*0.01
Min SpO2 (%), (SD)	88.0 (±3.5)	81.1 (±6.3)	*0.01
Mean SpO2 (%), (SD)	95.3 (±1.2)	93.4 (±1.5)	*0.01
Min Pulse (lpm), (SD)	55.09 (±10.2)	54.7 (±6.8)	0.9
max pulse (lpm), (SD)	100.5 (±9.0)	103.2 (±13.3)	0.39
Average pulse (lpm), (SD)	69.0 (±8.7)	69.0 (±7.6)	0.98

*Respiratory disturbance index (RDI), mean oxygen saturation (mean SpO2), minimum oxygen saturation (nadir SpO2), total time with oxyhemoglobin saturation below 90% (T90%), oxygen desaturation index (ODI-≥3%). SD, Standard deviation*.

*Statistically significant with p < 0.05 and highlighted in bold.

**Table 3:** Clinical characteristic between OSA and non OSA group of the complete cohort (*n* = 60).

**Variables**	**no OSA (*n =* 23)**	**OSA (*n =* 37)**	***p-*value**
Sex male, *n* (%)	10 (43.4)	22 (59.4)	0.35
Age (years), (SD)	38.3 (±12.1)	51.4 (±11.1)	*0.01
**COVID-19 severity**			
Mild, *n* (%)	12 (52.1)	6 (16.2)	*0.01
Moderate, *n* (%)	6 (26)	11(29.7)	0.41
Severe/critical, *n* (%)	5 (21.7)	20 (54)	*0.01
ICU stay, *n* (%)	7 (30.4)	27 (72.9)	*0.01
**Sleep questionnaire**			
ESS (points), (SD)	7.5 (±4.8)	9.5 (±5.6)	0.15
STOP-BANG (points), (SD)	2.2 (±1.3)	3.8 (±1.9)	*0.01
**Tobacco status**			
Current, *n* (%)	14 (60.8)	23 (62.1)	0.24
Former, *n* (%)	5 (21.7)	3 (8.1)	
Never Smoker, *n* (%)	4 (17.4)	11 (29.7)	
**Alcohol usage**			
Never, *n* (%)	11 (47.8)	14 (37.8)	0.44
Occasionally, *n* (%)	12 (52.1)	21 (56.7)	
Frequently, *n* (%)	0	2 (5.4)	
**Scholar years** ***n*** **(%)**			
<8 years, *n* (%)	4 (17.4)	17 (45.9)	0.07
8-12 years, *n* (%)	8 (34.7)	7 (18.9)	
>12 years, *n* (%)	11 (47.8)	13 (35.1)	
Rural area, *n* (%)	0	6 (16.2)	*0.04
**Anthropometry**			
BMI (Kg/m2), (SD)	30.3 (±4.8)	31.5 (±5.05)	0.37
Neck circumference (cm), (SD)	39.9 (±4.1)	43.0 (±5.5)	*0.01
Waist circumference (cm), (SD)	97.6 (±11.4)	107.3 (±12.2)	*0.01
Waist to hip ratio, (SD)	0.91 (±0.08)	0,97 (±0.06)	*0.01
**Comorbidities**			
Obesity (>30 K/m2), *n* (%)	11 (47.8)	23 (62.1)	0.20
IR at baseline, *n* (%)	1(4.3)	10 (27)	*0.02
T2DM at baseline, *n* (%)	3 (13)	4 (10.8)	0.54
Arterial hypertension, *n* (%)	6 (26)	13 (35.1)	0.33
SBP (mmHg), (SD)	124.7 (±15.1)	135.2 (±19.1)	*0.02
DBP (mmHg), (SD)	72.7 (±13.9)	76.9 (±13.2)	0.25
Dyslipidemia, *n* (%)	14 (60.8)	25 (67.5)	0.39
High LDL, (SD)	113 (±31.1)	117 (±46.3)	0.70
Low HDL, (SD)	46.4 (±20.8)	55.3 (±16.3)	0.09
Cholesterol (mmol/dL), (SD)	186.5 (±44,4)	202.1 (±60.5)	0.25
HDL (mmol/dL), (SD)	47.7 (±21.3)	54.7 (±16.1)	0.18
Triglycerides (mmol/dL), (SD)	149.3 (±132.4)	176.1 (±119.0)	0.43
Glycemia, (SD)	123 (±61.0)	111 (±31.6)	0.38
HOMA-IR, (SD)	5.5 (±5.9)	9.0 (±18.8)	0.3

*SDB, Sleep Disorder Breathing; COVID-19, Coronavirus 2019; ICU, Intensive care unit; ESS, Epworth sleepiness scale; BMI, Body mass index; IR, Insulin resistance; T2DM, Type 2 diabetes mellitus; SBP, Systolic blood pressure; DBP, Diastolic blood pressure; HDL, High density lipoproteins; HOMA-IR, homeostasis model assessment-estimated insulin resistance; SD, Standard deviation*.

*Statistically significant with p < 0.05 and highlighted in bold.

### Metabolic Changes After COVID-19 (Visit 1)

Regarding metabolic changes in subsets with OSA, 25/38 (67.5%) had dyslipidemia, 23/38 (60.5%) reported hypertriglyceridemia. Regarding to high LDL and low HDL, these presented values of 117 (±46.3) and 55.3 (±16.3) respectively. Respecting fasting glucose, the OSA group showed 111 (±31.6). HOMA-IR was 7.69 (±15.2), and 17/28.3%) reported a L/S ratio <1.0. A summary of the laboratory parameters is shown in Table 3.

### Cytokines Levels Among OSA Groups (Visit 1)

**Figure 2:**
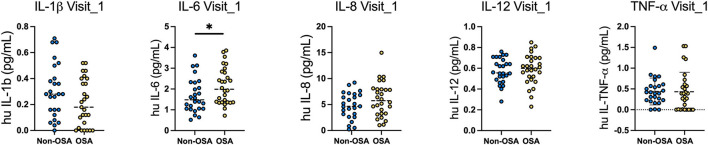
Levels of circulating cytokines in OSA and non-OSA patient groups at 4-months and 12-months after COVID-19 infection. Circulating levels of IL1b, IL-6, IL-8, IL-12 and TNF-a were measured in the serum of OSA and non-OSA at 4-months (Visit_1) and 12-months (Visit_2) after COVID-19 infection. Mann Whitney tests were used to analyze each cytokine and *p = 0.04.

A significant difference of circulating levels of IL-6 between groups was revealed 4-months after infection [non-OSA 1.52 (±0.70) vs. OSA 2.16 (±0.96)]. No significant differences between groups were observed for other cytokines or times points. Moreover, we observed that the OR of increased IL-6 was 2.54 (95%-CI, 1.21–5.32, *p* = 0.013). The subgroup of moderate to severe OSA also presented an increased risk of IL-6, 1.81 (±0.97) vs. 2.20 (±0.70) in the OSA group (Figure 2).

### Changes Between Visit 1 and Visit 2 Between OSA and Non-OSA Patients

**Figure 3:**
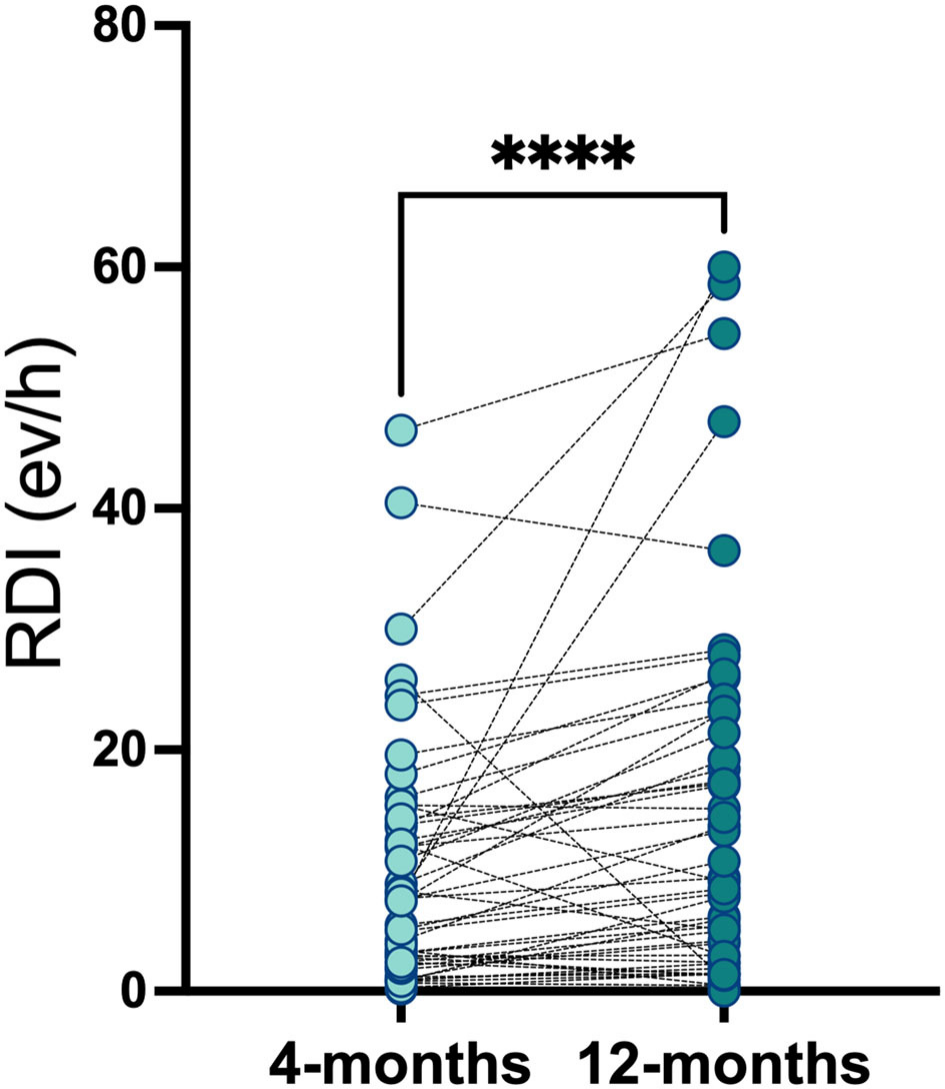
Respiratory disturbance index in OSA and non-OSA patient groups at 4-months and 12-months after COVID-19 infection. Respiratory disturbance index was measured in OSA and non-OSA at 4-months (Visit_1) and 12-months (Visit_2) after COVID-19 infection. Wilcoxon matched-pairs signed rank test was used and ****p < 0.0001.

A total of 57 (95%) participants completed the second follow-up (36 OSA and 21 non-OSA participants). Compared to first HSAT, one participant was re-scored as non OSA and 2 as OSA. There were no differences between the RDI scored at Visit 1 and Visit 2 (Figure 3). The OSA group reported an increased incidence of uncontrolled HT (50% in OSA in OSA vs. 27%in non OSA, *p* = 0.14), IR (85.7% in OSA vs. 57.7% in non-OSA, *p* = 0.046), and T2DM (32.1% in OSA vs. 11.1% in non OSA, *p* = 0.14). Among OSA population, DLCO test remained abnormal at Visit 2 in the 25% of the patients vs. 3.6% in non-OSA group (*p* = 0.02). Conversely, we found no difference regarding distance in 6MWT [median 474 mts (IQR, 417–542) in OSA vs. 510 mts (IQR, 440–582) in non OSA groups] (*p* = 0.368) and forced expiratory velocity at first second (FEV_1_) <70% (3.57% in OSA vs. 3.45% in non OSA) groups.

At 1 year of follow-up, we found no significant differences regarding mental and sleep health across groups (Table 4). Additionally, we found a significant decrease in the MOCA score among the OSA group, median 25.0 points (IQR, 21–27) in on-OSA vs. 22.0 points (IQR, 20.8–23) in OSA, *p* = 0.02. Regarding changes between Visit 1 and 2, the OSA group reported an increment in their RDI, median 2.8 ev/h (IQR, −0.6;7.5), p <0.01, and the non-OSA group improved the SF-12 Mental Score in median 4.0 points (IQR: −1.0;9.0), *p* = 0.03 (Table 5).

**Table 4:** Sleep and mental evaluation 1 year after COVID-19 infection.

**Variables**	**Non OSA (*n =* 21)**	**OSA (*n =* 36)**	***p-*value**
**Actigraphy**			
TST (minutes), (IQR)	380 (300–456)	335 (279–419)	0.24
Sleep Efficiency (%), (IQR)	88 (84.2–91.2)	85.7 (82.7–92.3)	0.83
WASO (minutes), (IQR)	39 (27.0–57.0)	46.0 (32–55)	0.95
**Sleep Questionnaires**			
ISI (points), (IQR)	12.0 (5.0–15)	7.5 (2.0–14)	0.24
SATED (points), (IQR)	7.0 (5.0–9.0)	8.0 (6.0–10)	0.22
ESS (points), (IQR)	12.5 (5.75–15.2)	8.5 (4.0–16.2)	0.58
**Mental and Physical health**			
BECK (points), (IQR)	7.0 (2.7–18.0)	9.0 (3.7–12.5)	0.99
HDAS-A(points), (IQR)	10.0 (8.0–11.2)	8.5 (7.0–10.2)	0.49
HDAS-D(points), (IQR)	10.5 (8.0–12.0)	9.0 (7.0–11)	0.09
SF12-Mental(points), (IQR)	45.5 (40–50)	48.0 (40–56.2)	0.46
SF12-Physical (points), (IQR)	51.0 (40.5–55)	43.0 (36.5–47)	0.07
MOCA (points), (IQR)	25.0 (21–27)	22.0 (20.8–23)	*0.02

*TST, Total sleep time; WASO, wake after sleep onset; SATED, Satisfaction, alertness, timing, efficiency, and duration; ISI, Insomnia Severity; ESS, Epworth Sleepiness Scale; BECK, Beck Depression Inventory; HADS, Hospital Anxiety and Depression Scale; MOCA, Montreal cognitive assessment. Values are expressed as median*.

*Statistically significant with p <0.05. and highlighted in bold.

**Table 5:** Changes between 4 months and 1 year after COVID-19 infection.

**Variables**	**Non OSA (*n =* 21)**	**OSA (*n =* 36)**	***p-*value**
**Anthropometry**			
Δ BMI(Kg/m), (IQR)	0.5 (−0.3;1.85)	1.4 (−0.2;2.8)	0.31
Δ Hip(cm), (IQR)	2.0 (0.5;6.0)	3.0 (−1.5;6.0)	0.69
Δ Neck Circumference (cm), (IQR)	3.0 (−0.5;3.5)	2.0 (−2.5;3.0)	0.43
**HSAT**			
Δ RDI (ev/h), (IQR)	−0.60 (3;1.5)	2.8 (−0.6;7.5)	*0.01
Δ T90(%), (IQR)	0.0 (−1.0;1.0)	1.0 (−1.5;8.0)	0.26
Δ Min Spo2	−2.0 (−5.0;5.0)	2.0 (−1.2;6.0)	0.11
Δ ODI-3%	2.0 (0.5;6.0)	3.0 (−1.5;6.0)	0.28
**Questionnaires**			
Δ HDAS-A (points), (IQR)	2.0 (−0.5; 5)	3.0 (−0.5;4)	0.82
Δ HDAS-D (points), (IQR)	4.0 (1.0;8.5)	5.0 (2.5;6.5)	0.83
Δ ISI (points), (IQR)	0.5 (−4.0;3.0)	1.0 (−3.5;1)	0.50
Δ BECK (points), (IQR)	1.0 (3.7;4.7)	1.0 (−6;4)	0.84
Δ SF12-Mental (points), (IQR)	4.0 (−1.0;9.0)	0.5 (−7.5;5.0)	*0.03
Δ SF12-Physic (points), (IQR)	1.0 (−4.25;5.0)	0.0 (−1.2;9.0)	0.43

*RDI, Respiratory disturbance index; T90%, Total time with oxyhemoglobin saturation below 90%; nadir SpO2, Minimum oxygen saturation; ODI-≥3%, Oxygen desaturation index; HADS, Hospital Anxiety and Depression Scale; ISI, Insomnia Severity; BECK, Beck Depression; SD, Standard deviation*.

*Statistically significant with p <0.05 and highlighted in bold.

### Risk of Incident Symptoms Among OSA Patients

The summary of the non-adjusted and fully adjusted logistic regression analysis is show in Table 6. In the non-adjusted model, we found an association between OSA and DLCO <80%, OR: 9.3 (95%-CI 1.5–181, *p* = 0.04), IR, OR: 4.4 (95%-CI, 1.2–18.2, *p* = 0.02), and abnormal MOCA score, OR: 2.9 (95%-CI, 1.85–11.3, *p* = 0.04). We also found a significant interaction between OSA and development of ARDS during the acute phase (*p* = 0.05). Those participants with OSA who develop ARDS reported an adjusted OR 20.4 (95%-CI, 1.04–504) risk of neurocognitive impairment.

**Table 6:** Multivariable model between OSA and risk of incident metabolic, pulmonary, and neurocognitive sequelae 1 year after COVID-19.

**Variables**	**Model 1 (OR, 95%-CI)**	***p-*value**	**Model 2 (OR, 95%-CI)**	***p-*value**
DLCO	9.3 (1.5–181)	*0.04	13.4 (0.6–182)	0.16
Insulin resistance	4.4 (1.2–18.2)	*0.02	2.5 (0.3–18.1)	0.33
Diabetes Mellitus	3.6 (0.93–18.1)	0.07	1.5 (0.2–10.5)	0.62
Uncontrolled HTA	2.6 (0.89–8.1)	0.08	1.8 (0.3–8.7)	0.45
MOCA	2.9 (1.85–11.3)	*0.04	4.2 (0.7–26)	0.10

*DLCO, Diffusing Capacity of The Lungs for Carbon Monoxide; MOCA, Montreal cognitive assessment. Additionally we performed a logistic regression analysis, as confounder analysis, we conducted an unadjusted an adjusted model by age, gender, BMI, development of ARDS during the acute infection. The values were reported as Odds ratios (OR) with a 95% confidence interval (95%-CI)*.

*Statistically significant with p <0.05. and highlighted in bold.

## Discussion

The main findings of our study described the association between OSA with long-term symptoms and inflammatory cytokines between 4-months and one-year follow-up after acute phase of COVID-19. We observed that OSA patients report an increased risk of glycemic changes, circulating levels of IL-6, neurocognitive impairment and persistent abnormal DLCO. Moreover, undiagnosed OSA is prevalent in this population, and is associated with an incident risk of worse outcomes during follow-up. Furthermore, our results were independent by delta weight within Visit 1 and 2.

In this study, we explored the changes in a wide burden of sequalae amongst survivors of COVID-19. Additionally, we hypothesized that patients with undiagnosed OSA would show increased circulating levels of IL-6 and therefore, would have an increased risk of metabolic, pulmonary, and neurocognitive sequelae. We hypothesized that the mechanisms of this association are related to the interaction between nocturnal hypoxemia, sympathetic activation, and a persistent proinflammatory state (34). Previous research has implicated sleep fragmentation and chronic intermittent hypoxia (CIH) during sleep as underlying mechanisms linking OSA to glycemic impairment through alterations in insulin sensitivity and glucose disposal (35). The presence of CIH increases several oxidative stress markers, causing endothelial and systemic inflammation (12, 13). Clinically, CIH increases the risk for metabolic dysregulation (9). Moreover, a recent publication showed an increased risk of mortality, and ICU admission in patients with a medical record of OSA, and an increased prevalence of undiagnosed SDB in patients with COVID-19^2^.

We also hypothesized that during the acute COVID-19 phase, the pro-inflammatory state, in addition to hypoxemia and the severity of the disease, pancreatic dysregulation can also be enhanced, increasing the risk of glycemic impairment at long term, and probably, the new onset of sequalae (30, 31). This study found a high prevalence of both IR and T2DM among OSA patient, 4-months of follow-up, and after 1 year. This population also reports an increased prevalence of other major cardiovascular factors, such as dyslipidemia and HT.

Another relevant finding is the association between undiagnosed OSA and neurocognitive impairment, reporting a significant interaction with ARDS after the acute phase (36, 37). Moreover, this association was higher among OSA participants who develop ARDS. The risk of neurocognitive impairment after COVID-19 was previously reported as a sequela (37). However, the impact of OSA over these symptoms is not well known. Previous studies suggest that both OSA and COVID-19 increase the expression of several inflammatory stress, including proinflammatory cytokines as IL-6, developing an endothelial vulnerability, and therefore, increasing the permeability of this markers to the brain (38). In fact, inflammation and endothelial dysfunction observed in OSA patients have been associated with a reduction of vascular elasticity and increase coagulation, promoting atherosclerosis. All these elements can lead to cellular dysfunctions in several organs such as the heart and the brain (39). Similarly, OSA has been related to Alzheimer's disease and advancing cognitive decline in elderly population, mainly by intermittent hypoxia and sleep dysregulation (40). According to a systematic review and meta-analysis, individuals with SDB are 26% more likely to develop cognitive impairment (41). Furthermore, the apneic events occur during both REM and non-REM sleep consequently generating a decrease of sleep phases (42). These alterations on architecture sleep have been correlated with cognitive skills impairment (39). Furthermore, several studies have observed neurocognitive impairment, PSTD, sleep disorders psychiatric comorbidity and poor QoL in COVID-19 survivors (37, 43, 44). We believe that our findings are useful to improve health-based strategies, focused on identifying the population with an increased risk of neurocognitive impairment, especially in those who developed long-lasting symptoms due to COVID-19, in addition to a clinical history of OSA.

Regarding the impact of COVID-19, in our study, we found no difference regarding the HSAT, and probably, HSAT can be performed from the 4 to 1 year after hospital discharge, providing a good opportunity to identify population with OSA to prevent risk of long-term symptoms. In our study, the association between ICU admission and COVID-19 severity (ARDS development), was also associated with persistent abnormal DLCO after 1 year. Further research exploring this association's strength, and the potential effect of nocturnal hypoxemia should improve this finding. Although this study reported several important findings, there were limitations associated with it. These include a small sample size, and the limited number of events of outcomes during the follow-up. However, the prevalence of diabetes and other comorbidities at baseline was higher than the current prevalence in a similar age group in Chile. Further research, including longer follow-up, is also required. Population-based studies are also necessary to confirm our results. We also used a fasting glucose exam, and HbA1c, following current ADA recommendations, however, we limited our analysis to this method. Other methods to diagnose T2DM, such as the oral glucose tolerance test or glycated hemoglobin levels, should be included to rule out T2DM. We performed an HSAT as this study is recommended in populations with a high pretest probability of OSA, however, the gold standard remains the polysomnography (PSG) (30). We were unable to use the PSG due to the sanitary restrictions, and the lack of PSG availability during the COVID-19 pandemic. HSAT is an excellent alternative for the diagnosis of a population with OSA (45). Finally, this study was performed during sanitary restrictions, and concern about residual confounders such as lack of physical activity and weight gain during this period can overestimate this association's strength.

## Data Availability Statement

The original contributions presented in the study are included in the article/Supplementary Material, further inquiries can be directed to the corresponding author.

## Ethics Statement

All procedures performed in this study were in accordance with the ethical standards of the institutional and/or national research committee from Servicio de Salud Bio Bio (IRB: CEC113), and Servicio de Salud Concepcion (IRB: CEC-SSC: 20-07-26), Chile. And with the 1964 Helsinki declaration and its later amendments or comparable ethical standards. The patients/participants provided their written informed consent to participate in this study.

## Author Contributions

Study design and ISRCTN register: GL. Patient recruitment, sample processing, and data analysis: SS, CC, RQ, BA, LL, FZ, VO, EN-L, MH-B, AT, DC, JG, GH, and GL. Interpretation of the results: GL, EN-L, GH, AT, FB, JL, and DE. Manuscript preparation: GL, JL, DE, SS,CC, and EN-L. Editing and approval: GL, LL, FZ, MH-B, DE, JL, AT, EN-L, FB, SS, CC, JG, RQ, and BA. All authors approved the final manuscript.

## Funding

This study was supported by the Agencia Nacional de Investigación y Desarrollo (ANID, COVID1005), Chilean Government. GL declares funding for research by the American Academy of Sleep Medicine (AASM, 254-FP-21). JG, FB, and AT declare funded by ISCIII (CIBERESUCICOVID, COV20/00110). EN-L, SS, CC, RQ and BA were funded by Fondecyt 1211480 and COVID-19 Genomics Network (C19-GenoNet) ACT210085. Figure 1 was created with BioRender.com and Flow Cytometer was funded by EQM150061 (FONDEQUIP-ANID).

## Conflict of Interest

The authors declare that the research was conducted in the absence of any commercial or financial relationships that could be construed as a potential conflict of interest.

## Publisher's Note

All claims expressed in this article are solely those of the authors and do not necessarily represent those of their affiliated organizations, or those of the publisher, the editors and the reviewers. Any product that may be evaluated in this article, or claim that may be made by its manufacturer, is not guaranteed or endorsed by the publisher.

## Footnotes

^1^ https://www.worldometers.info/coronavirus/

^2^ https://www.paho.org/chi/
