# Supplementary figures and images for: Impact of Obstructive Sleep Apnea (OSA) in COVID-19 Survivors, Symptoms Changes Between 4-Months and 1 Year After the COVID-19 Infection
Source: Front Med (Lausanne). 2022 Jun 14;9:884218. doi: 10.3389/fmed.2022.884218 (PMC9237467; doi:10.3389/fmed.2022.884218)

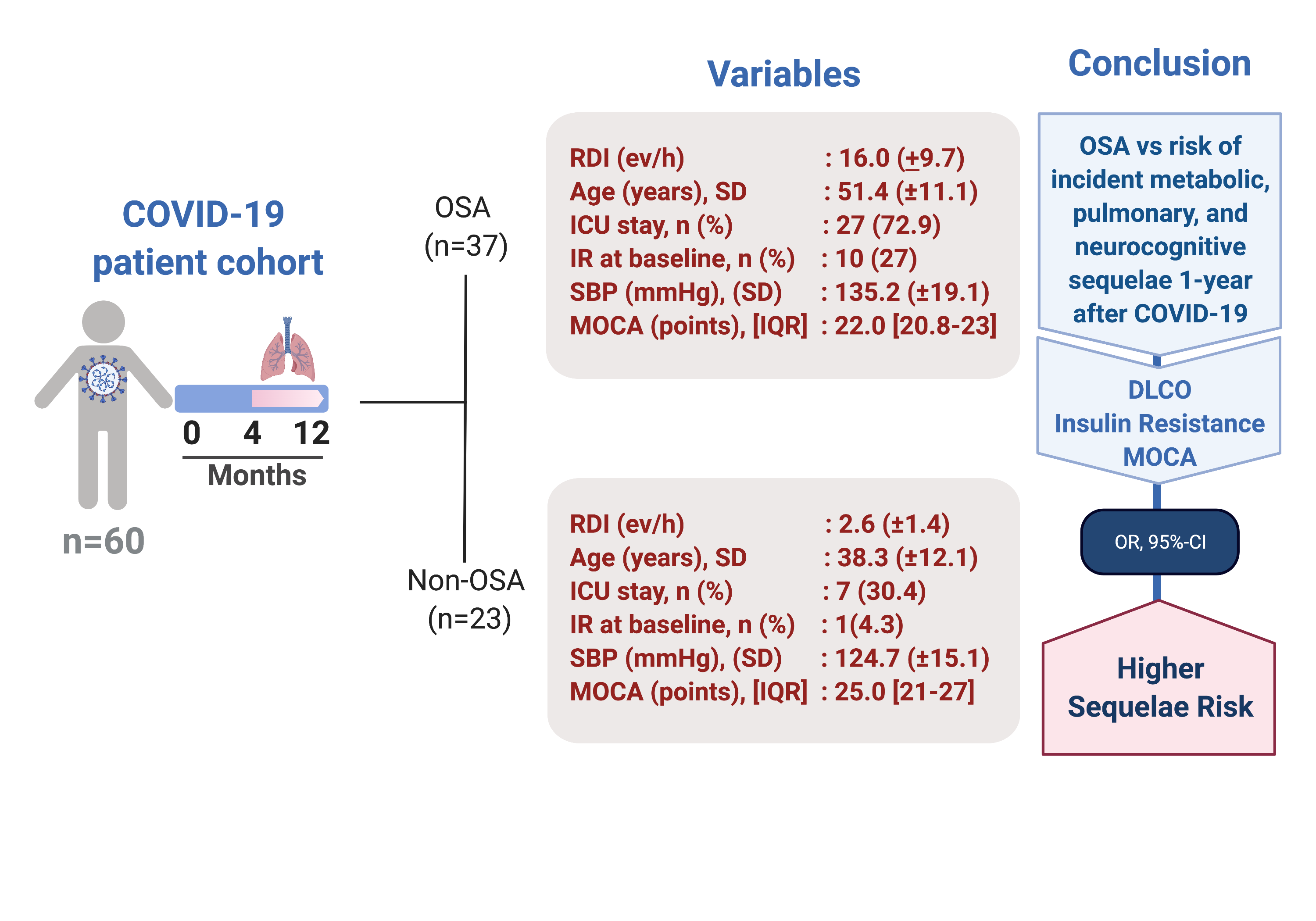

Supplement: Supplementary file 1 [file Image_1.PNG]
